# Domain-aware enzyme classification via multiple instance learning

**DOI:** 10.1093/bioinformatics/btag804

**Published:** 2026-10-30

**Authors:** Qingyang Xiao, Yuzhen Ye, Haixu Tang

**Affiliations:** Luddy School of Informatics, Computing and Engineering, Indiana University, Bloomington, IN 47408, United States; Luddy School of Informatics, Computing and Engineering, Indiana University, Bloomington, IN 47408, United States; Luddy School of Informatics, Computing and Engineering, Indiana University, Bloomington, IN 47408, United States

## Abstract

**Motivation:**

Accurate Enzyme Commission (EC) number prediction is fundamental to protein function annotation and metabolic pathway reconstruction. Recent methods based on protein language model embeddings or 3D structure have improved EC prediction, but important limitations remain. Whole-sequence embeddings can dilute local functional signals, such as catalytic residues and functional domains, whereas structure-based methods depend on accurate protein structures and are therefore difficult to apply broadly.

**Results:**

We present MIL-BC, a multiple instance learning framework for EC prediction that models each protein as a bag of domains rather than a single sequence-level object. This design is motivated by the observation that enzymatic function is often determined by constituent functional domains instead of the full-length sequence alone. Domain instances are generated automatically using our DCTdomain algorithm, avoiding dependence on curated domain annotations. MIL-BC aggregates domain-level evidence into protein-level EC predictions and assigns interpretable weights to domains that contribute most strongly to each prediction. We further combine the domain embedding with global embedding in MIL-BC+. MIL-BC+ achieved F1 improvements of 0.03–0.17 over the sequence-based baseline on all four test sets. The largest gains were observed for proteins with multiple enzymatic functions, where domain-level modeling better separates mixed functional signals. These results show that domain-centric multiple instance learning is an accurate and interpretable strategy for enzyme classification. By improving EC prediction without requiring structural data or curated domain labels, MIL-BC provides a scalable framework for large-scale protein function annotation.

**Availability and implementation:**

Code is available as a GitHub repository at https://github.com/omics-ai/enzyme-mil.git.

## 1 Introduction

Enzymes catalyze a vast array of biochemical reactions in living cells, driving essential biological processes ranging from DNA replication and signal transduction to the synthesis of vital molecules and the breakdown of nutrients for energy (Fruton 1999, Copeland 2023). To organize this immense functional space, the Enzyme Commission (EC) provides a hierarchical classification system, assigning a unique four-digit EC number to each enzyme based on the specific reaction it catalyzes (McDonald and Tipton 2023). The automated and accurate prediction of these EC numbers is a primary task in functional genomics. It is essential not only for annotating newly sequenced genomes (Kanehisa 2017) but also for understanding complex metabolic pathways and guiding efforts in drug discovery and synthetic biology, where precise functional knowledge of proteins is critical.

Conventional EC number prediction methods rely on protein homology search algorithms (e.g. BLAST; Claudel-Renard *et al.* 2003, Yin *et al.* 2012, Pearson 2015), which compare a query sequence to a database of annotated enzymes (e.g. UniProt; Morgat *et al.* 2020). While effective for close homologs, these methods often perform poorly on target proteins with low sequence similarity with the annotated enzymes, even if the function is conserved (Omelchenko *et al.* 2010, McDonald and Tipton 2014). To overcome this gap, machine learning (ML) methods were developed. Conventional ML approaches (Tan *et al.* 2019) exploited a feature extraction step to convert a protein’s sequence into a fixed-length numerical “feature vector.” These feature vectors were then used to train standard classifiers such as support vector machines, random forests, or neural networks. For example, EFICAz2.5 (Kumar and Skolnick 2012) achieved high-precision enzyme classification by integrating pairwise sequence alignment, profile hidden Markov models, and the recognition of conserved catalytic residues.

More recently, the field has been revolutionized by deep learning, which does not require manual feature engineering. These methods employed various deep neural network (DNN) architectures to learn the relevant features directly from the raw protein sequence (Kim *et al.* 2025). For example, DeepEC (Ryu *et al.* 2019) used convolutional neural networks (CNNs) as its major engine for EC number prediction and showed higher precision compared to conventional ML tools (Ryu *et al.* 2019). DEEPre (Li *et al.* 2018) employed a hybrid DNN model architecture that processes sequence-dependent features (e.g. PSSM) with a CNN followed by a long short-term memory, while processing sequence-independent vector features (like occurrences of functional domains) with a fully connected layer.

Some state-of-the-art models leverage pretrained protein language models, such as ESM-1b (Rives *et al.* 2021) and ESM-2 (Lin *et al.* 2022), to generate a single “sequence-level” embedding for the entire protein. CLEAN, for instance, developed a contrastive learning framework to assign EC numbers based on proteins’ ESM embedding vectors, achieving better accuracy and sensitivity compared to previous models (Yu *et al.* 2023). DeepES built an individual deep fully connected neural network for each reaction class (i.e. RClass) in KEGG (Kanehisa and Goto 2000) using the ESM-2 embedding of protein sequences (Hirota *et al.* 2025) as the input. More recent EC number prediction models, such as GraphEC (Song *et al.* 2024), utilized 3D structural information, either from experimental sources or high-quality predictions [by AlphaFold (Jumper *et al.* 2021) or ESMFold (Lin *et al.* 2022)], which is encoded via geometric graph learning and used in conjunction with sequence-level embeddings. Despite their relative success, these methods have limitations, and thus, there is still significant room for further improvement in enzyme classification. For instance, sequence-level embeddings may dilute the functional signal in enzymes by averaging it across the entire protein, while structure-based methods are computationally expensive and rely on the availability of high-fidelity protein structures.

A critical insight is that an enzyme’s function (i.e. the reaction and substrates it catalyzes) is often not determined by its entire polypeptide chain, but by the presence or combination of specific, modular functional units known as domains (Khosla and Harbury 2001), or even the key residues located within the specific domain(s) (Bartlett *et al.* 2002). The protein universe contains many multidomain proteins, in which domains are subunits that can fold and function independently (Yu *et al.* 2019). Accordingly, many enzymes are multidomain proteins, and their catalytic activity may be conferred by a single catalytic domain, while other domains are responsible for regulation, substrate binding, or localization. This modular nature suggests that a more robust representation for an enzyme is not a single embedding vector, but rather a “bag” of vectors encoding its constituent domains. This observation informs our proposed approach that considers the protein as a collection of functional domains, any one of which might be the key determinant of its enzymatic function (represented by its EC number).

This “bag-of-domains” representation, however, presents a distinct machine learning challenge: we have a label (i.e. the EC number) for the entire protein (the “bag”), but we do not know *a priori* which specific domain (the “instance”) is responsible for that function. This scenario is the classic use case for multiple instance learning (MIL; Carbonneau *et al.* 2018), which is a supervised learning approach for problems where labels are assigned to groups of instances (bags) rather than to individual instances. In the standard MIL assumption, a bag is labeled positive if it contains at least one positive instance, and otherwise, it is labeled negative. This maps perfectly to enzymatic function: a protein may possess a specific catalytic activity (a positive bag label) if it contains at least one domain (a positive instance) that confers that activity.

Building on this insight, we present a novel machine learning framework for enzyme classification by explicitly formulating the problem using MIL. We represent each protein as a “bag,” and its constituent protein domains as the “instances” within that bag. To generate these instances, we leverage an automated algorithm, “DCTdomain,” previously developed by us (Iovino *et al.* 2024a, 2024b), which first partitions a protein into domains (or subdomains) based on a contact matrix predicted directly from the input protein sequence, and then represents each domain as a vector. This process allows us to automatically encode any input protein sequence as a “bag-of-domains” without relying on known domain annotations. Using these inferred bags as input, we train an MIL model (MIL-BC) to predict the final EC number. The model learns to aggregate information from the instance-level domain embeddings, enabling it to identify the specific, functionally critical domains responsible for catalysis.

## 2 Materials and methods

As illustrated in Fig. 1, the workflow of the MIL algorithm consists of three essential components. First, an input protein sequence is encoded into a bag of domain embeddings using DCTdomain (Iovino *et al.* 2024a). Next, the domain embeddings within a bag are integrated into a single, fixed-length protein embedding using an attention mechanism (Vaswani *et al.* 2017). This attention layer learns to assign different importance weights to each domain, allowing the model to focus on the most functionally relevant domains. This integrated protein embedding is then fed into a multilayer perceptron (MLP) that uses a binary cross-entropy (BCE) loss for each EC number, treating the task as a multilabel problem (e.g. predicting 1 for the correct four-digit EC number and 0 for all others). Finally, a contrastive learning algorithm is applied to refine the integrated protein embedding, which is subsequently used to infer the EC number of any given protein sequence by applying a *k*-nearest neighbor (KNN) search against the embedding vectors of all training samples.

**Figure 1:**
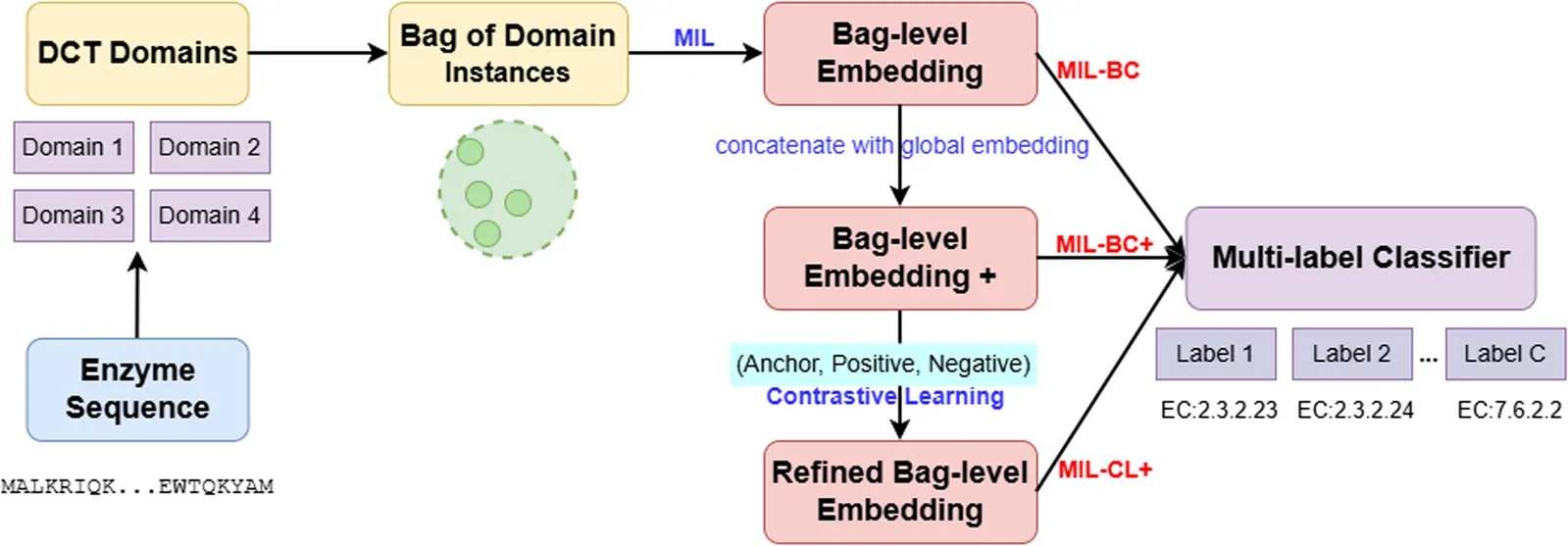
Our MIL framework for EC number prediction consists of three components: (i) Converting the input protein sequence into a bag of domain embeddings, (ii) generating a bag-level embedding using an MIL attention layer, and (iii) refining this bag-level embedding through CL. From this framework, we implemented and evaluated three distinct predictors: *MIL-BC*, which uses the bag-level embedding directly; *MIL-BC+*, which uses a combined input of the bag-level embedding by DCTdomain and a global protein-level embedding by ESM-2; and *MIL-CL+*, which uses the refined embedding by CL.

### 2.1 Formulation of the problem

Formally, let $P=\{P_{1},P_{2},\ldots ,P_{N}\}$ be a dataset of *N* proteins, and $Y=\{Y_{1},Y_{2},\ldots ,Y_{N}\}$ be their corresponding four-digit EC number. In our study, we consider the set of C=5242 EC classes, belonging to the seven top-level of EC numbers, indicating seven general classes of enzymes, Oxidoreductases (‘1’), Transferases (‘2’), Hydrolases (‘3’), Lyases (‘4’), Isomerases (‘5’), Ligases (‘6’), and Translocases (‘7’), respectively. Our goal is to predict multiclass classification, where $Y_{i}$ belongs to one of *C* EC classes.

In the MIL framework, each protein $P_{i}$ is not treated as a single data point, but as a bag $B_{i}$ containing a variable number of instances. In this context, the instances are the protein’s constituent domains. The core MIL assumption is that the bag-level label $Y_{i}$ (the EC number) is determined by the properties of the instances (domains) within the bag. We assume a positive label (a specific EC class) exists if at least one domain instance within the protein is responsible for that catalytic function.

### 2.2 Automated domain embedding

To convert a protein sequence *P* into a bag of domain embeddings, we utilized the DCTdomain algorithm (Iovino *et al.* 2024a). This process begins by using the pretrained protein language model ESM-2 (Lin *et al.* 2022) to generate residue-level embeddings of the input sequence and a predicted contact map C between the residues. The predicted contact map is used for partitioning the protein into structural domains (or subdomains) with the minimum contacts between the domains. Domains can be either continuous or discontinuous in sequence (Iovino *et al.* 2024a), with the former being the majority.

For each domain, we generate a single contextual vector from the embeddings of all the residues in that domain. First, we extract the corresponding $L_{j}$ residue-level embeddings to form an embedding matrix of $L_{j}\times V$. This input matrix is then subjected to dimension reduction as follows: 2D discrete cosine transformation (DCT) is first applied to the input embedding matrix to compute frequency coefficients, and then an inverse DCT (iDCT) is applied to only low-frequency coefficients (discarding the high-frequency ones) to produce a dimension-reduced matrix (Ahmed *et al.* 1974). The compressed matrix is then flattened to produce a 1D vector (referred to as a DCT fingerprint). In this way, a domain (of any length) is represented as a vector of fixed dimension (e.g. 480). We previously showed that ESM-2 contact map-based domain predictions gave good performance for similarity detection between proteins based on domain-level embeddings (Iovino *et al.* 2024a).

### 2.3 Multiple instance learning using binary classes

Following domain embedding, each domain *j* of a protein *i* (for i=1,…,N) is encoded into a *D*-dimensional feature vector, $d_{i,j}\in R^{D}$. Each protein $P_{i}$ is thus represented by a bag of its $K_{i}$ domain embeddings:


$$B_{i}=\{d_{i,1},d_{i,2},\ldots ,d_{i,K_{i}}\}$$

where $K_{i}$ is the number of domains, which can vary for each bag.

The MIL model consists of three main components: an instance transformation network, an attention-based aggregation layer, and a final classification network. To increase model capacity, the *D*-dimensional domain embeddings $d_{i,j}$ are first projected into a new *M*-dimensional feature space (M=480) using a shared instance-level network, implemented as an MLP with one fully connected layer. The output of this network is an M–dimensional embedding: $h_{\theta_{h}}$:


$$h_{i,j}=h_{\theta_{h}}(d_{i,j})=\text{ReLU}(W_{h}d_{i,j}+b_{h})$$

where $W_{h}\in R^{M\times D}$ and $b_{h}\in R^{M}$ are learnable parameters. This step yields a new bag of transformed instances $B_{i}^{\prime }=\{h_{i,1},\ldots ,h_{i,K_{i}}\}$, where $h_{i,j}\in R^{M}$.

The key objective of MIL is to aggregate the variable number of domain (instance) embeddings $B_{i}^{\prime }$ into a single, fixed-length vector $z_{i}$ that represents the entire protein (bag). We use a learnable attention mechanism $g_{\theta_{a}}$ to perform a weighted sum, allowing the model to identify the most functionally relevant domains. The attention mechanism computes an unnormalized score $e_{i,j}$ for each domain instance $h_{i,j}$:


$$e_{i,j}=w^{T}\text{tanh}(Vh_{i,j}+b_{V})$$

where $V\in R^{L\times M}$ and $b_{V}\in R^{L}$ are parameters of a linear layer, and $w\in R^{L}$ is a learnable parameter vector. The tanh function serves as a nonlinear activation. The set of parameters $\theta_{a}=\{V,b_{V},w\}$ is shared across all domains (instances). These scores are then normalized across all domains in the protein using the softmax function to obtain the attention weights $a_{i,j}$:


$$a_{i,j}=\frac{\;\text{exp}(e_{i,j})}{\sum_{k=1}^{K_{i}}\;\text{exp}\;(e_{i,k})}$$

Each $a_{i,j}$ represents the learned “importance” of domain *j* in contributing to the protein’s final label. The final protein embedding $z_{i}\in R^{M}$ is computed as the weighted average of the domain embeddings:


$$z_{i}=\sum_{j=1}^{K_{i}}a_{i,j}h_{i,j}$$

Notably, the vector $z_{i}$ is permutation-invariant, meaning the order of domains in the bag does not affect the final protein representation.

Finally, the fixed-length protein embedding $z_{i}$ is fed into a final classification network $c_{\theta_{c}}$, implemented by an MLP followed by a softmax activation, to predict the probability distribution over a total of *C* EC classes.


$${\hat{Y}}_{i}=c_{\theta_{c}}(z_{i})=\text{softmax}(W_{c}z_{i}+b_{c})$$

where $W_{c}\in R^{C\times M}$ and $b_{c}\in R^{C}$ are the parameters of the final classification layer.

To explicitly model the global protein features alongside local domain information, we developed another approach (denoted as *MIL-BC+*), where the final fixed-length protein embedding $z_{i}$ was formed by concatenating two vectors: the aggregate domain embedding (generated by the MIL attention layer) and the ESM-2 embedding vector of the entire protein sequence. This combined representation, which more than tripled the final feature dimension (e.g. from 480 to 1760), was then fed into the classification network.

The entire model, with all parameters $\theta =\{\theta_{h},\theta_{a},\theta_{c}\}$, is trained end-to-end by minimizing the BCE loss L over the training set:


$$L(\theta )=-\frac{1}{N}\sum_{i=1}^{N}\sum_{c=1}^{C}Y_{i,c}\;\text{log}({\hat{Y}}_{i,c})$$

where $Y_{i,c}$ indicates the ground-truth label of protein *i* (1 if it belongs to class *c*, 0 otherwise), and ${\hat{Y}}_{i,c}$ is the model’s predicted probability for that class.

### 2.4 Contrastive learning for latent space refinement

While a classifier trained with a BCE loss can predict EC numbers, this objective function primarily focuses on maximizing the probability of the correct class. It does not explicitly enforce a geometric structure on the latent space. This can be problematic for EC classification, where many classes are functionally similar yet distinct (e.g. subclasses within the same main class). As revealed in previous studies (Yu *et al.* 2023), contrastive learning (CL) addresses this issue by directly optimizing the similarity structure of the embedding space, aiming to learn a latent representation where proteins from the same EC class are “pulled” together, while proteins from different classes are “pushed” apart.

We introduce a projection head, $f_{\theta }$, implemented in an MLP network, which maps the aggregate protein embedding z (generated by the MIL-attention layer) into a new *P*-dimensional contrastive latent space: $h=f_{\theta }(z)$. This projection head $f_{\theta }$ is trained via CL using a triplet margin loss. Following the approach adopted by CLEAN (Yu *et al.* 2023), we form training triplets $\left(z_{i},z_{j},z_{k}\right)$ from the training data, where $z_{i}$ is the “anchor” embedding (from a reference enzyme in a given class), $z_{j}$ is the positive embedding (from an enzyme in the same EC class as the anchor) and $z_{k}$ is the negative embedding (from an enzyme in a different EC class). These are projected into the latent space via the projection head: $h_{i}=f_{\theta }(z_{i})$, $h_{j}=f_{\theta }(z_{j})$, and $h_{k}=f_{\theta }(z_{k})$.

The model’s parameters θ are optimized by minimizing the triplet margin loss: $L_{CL}$, where a fixed number of positives are sampled from the same EC class as the anchor, and a fixed number of negatives are hard-mined


$$L_{CL}=\sum_{e\in E}\frac{-1}{\left|P(e)\right|}\sum_{h_{p}\in P(e)}\;\text{log}\;\frac{\;\text{exp}(h_{e}\cdot h_{p}/\tau )}{\sum_{h_{a}\in A(e)}\;\text{exp}\;(h_{e}\cdot h_{a}/\tau )}$$

where *E*, P(e) and A(e) represent the entire set of enzymes, the positive set of enzymes (i.e. those in the same class as the enzyme *e*), and the sampled anchor set, respectively. This loss function penalizes the model unless the distance to the positive sample is smaller than the distance to the negative sample by at least the margin $\alpha =e^{\tau }$.

### 2.5 Datasets

We utilized the same training set as CLEAN (Yu *et al.* 2023), which was collected from UniProt (UniProt Consortium 2021). During training, we removed enzyme sequences shorter than five amino acids, which excluded two sequences. Consequently, the training set contained 227 360 enzyme sequences associated with 5242 unique EC numbers. The dataset was divided into five folds for five-fold cross-validation (CV), following the exact splits provided by CLEAN (Yu *et al.* 2023), which contained 45 150, 45 131, 45 142, 45 156, 45 151 protein sequences, respectively.

We employed four independent test sets for evaluation: New-392, Price-149, Uniprot25-all, and Uniprot25-multi. The first two are widely used benchmarks. New-392 (Yu *et al.* 2023) contains 392 enzyme sequences (386 unique) with 177 EC numbers that were absent from the training data at the time of its creation (April 2022). Price-149 (Price *et al.* 2018) is an independent set of 149 unique enzyme sequences. The third test set Uniprot25-all was constructed by us from 987 unique enzymes deposited in UniProt (May 2022 to October 2025), which had no overlap with the training data, New-392, or Price-149. Based on Uniprot25-all, we created a subset named Uniprot25-multi that contains only sequences with multiple enzymatic functions. All the datasets are available on Zenodo: https://doi.org/10.5281/zenodo.22598911

A substantial fraction of enzymes is associated with multiple EC numbers, reflecting their ability to catalyze more than one biochemical reaction. In our 227 360-protein training set, 10 583 (4.7%) have two EC labels, 1044 (0.5%) have three, and 296 (0.1%) have four or more. Predicting the complete set of EC classes for these multifunctional proteins is particularly challenging. The Uniprot25-multi dataset enables systematic comparison of model performance on multifunctional enzymes. Full dataset statistics are shown in Table 1.

**Table 1:** The statistics of the four independent test sets.

	Train	New-392	Price-149	Uniprot25-all	Uniprot25-multi
Total	227 360	392	149	987	77
Unique	191 458	386	149	987	77
Label = 1	215 437	334	146	910	0
Label = 2	10 583	35	3	68	68
Label > 2	1340	23	0	9	9

To rigorously evaluate model performance while preventing information leakage, we constructed leakage-free evaluation sets following the protocol established by EC-Bench (Davoudi *et al.* 2025). Specifically, we merged all samples from three independent test sets (New-392, Price-149, and Uniprot25-all) into a single candidate pool. Protein sequences from both the training data and this test pool were then clustered using MMseqs2 (Steinegger and Söding 2017) at sequence identity thresholds of 70% and 50%. Any test sequence that clustered with a training sequence at the specified threshold was removed. This procedure resulted in two evaluation sets with no sequence overlap above the specified identity thresholds, referred to as Test-70 and Test-50, which enable robust assessment under increasingly stringent homology constraints. The Test-70 set contains 1057 enzyme sequences, while Test-50 contains 732 sequences.

### 2.6 Implementation and evaluation

All the domain embeddings were extracted by DCTdomain (Iovino *et al.* 2024a) and global embeddings were generated by ESM-2. For each enzyme sequence, both representations can be generated within seconds. This preprocessing is performed once, offline. Our MIL framework consists of lightweight fully connected layers combined with a gated attention pooling mechanism (Ilse *et al.* 2018), which adaptively weights the contribution of individual domain embeddings while maintaining low computational complexity. We implemented the MIL framework in PyTorch. Specifically, we implemented three prediction models. The first two, MIL-BC (BC stands for Binary Classification) and MIL-BC+ (where + denotes the integration of a global embedding), are trained using a BCE loss. MIL-BC utilizes the aggregated bag-level embedding alone, while MIL-BC+ concatenates this with a global protein embedding (derived from ESM-2). To account for multifunctional proteins, both models predict all EC numbers that receive a classification score above a defined threshold (0.5 by default). The third model, MIL-CL+, employs a CL framework to refine the embedding space, and then predicts EC numbers via a KNN search, assigning labels based on the most similar enzymes in the training set within this refined latent space. During MIL-CL+ training, we adapted the data augmentation strategy from CLEAN (Yu *et al.* 2023) for the orphan EC classes (i.e. those containing only one protein sequence in the training set) to improve the prediction for these sequences. Finally, to isolate the specific contribution of the MIL framework, we established a baseline model: ESM2-BC. This model relies solely on the global ESM-2 embedding and is trained with the same BCE loss, allowing for a direct performance comparison against the MIL-enhanced architectures.

We trained all models on a single NVIDIA H100 GPU. MIL-BC was trained for 80 epochs with a batch size of 512 using the AdamW optimizer, a learning rate of 0.0002, and a weight decay of 0.01. MIL-CL+ was trained for 1500 epochs with the Adam optimizer and a learning rate of 0.0005. For MIL-BC and MIL-BC+, we applied a postprocessing strategy (adjust the default 0.5 cutoff threshold used in BCE) to ensure that each sample received at least one valid label. The implementation, along with the training and test sets, are released as open-source software on GitHub (https://github.com/omics-ai/enzyme-mil.git).

All models were evaluated against state-of-the-art protein sequence-based EC number prediction models, including DeepEC (Kim *et al.* 2023), CLEAN (Yu *et al.* 2023), and EnzHier (Duan *et al.* 2025), through both CV and on the four independent datasets used by previous benchmarking studies. We employed three common metrics, precision, recall, and F1 score, respectively, to compare model performance. In addition to the models’ F1 score on predicting all four digits of EC numbers, we computed their F1 scores on predicting each of the four levels.

## 3 Results

### 3.1 Bag-of-domains representations of enzymes

We first analyzed the structural and functional characteristics of the proteins in our training set. Figure 2 illustrates their length distribution and the distribution of the number of domains per enzyme, showing a wide range from single-domain to complex multidomain proteins. The proteins in the independent testing datasets showed the same trends (as shown in Table 1).

**Figure 2:**
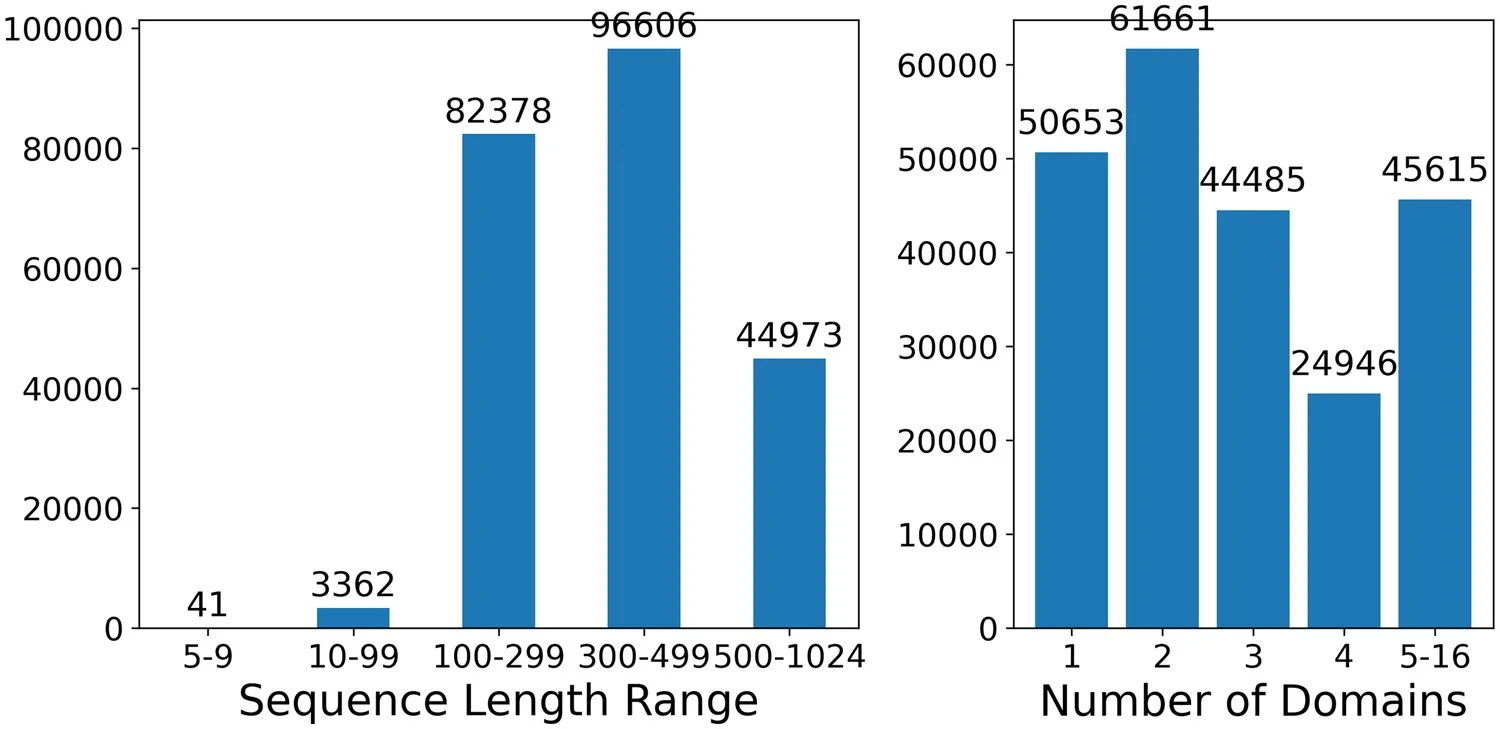
Distributions of protein length (left) and domain count per enzyme (right) for all proteins in the training set.

We observed a strong correlation between this structural complexity and functional multiplicity, where multifunctional enzymes (i.e. those assigned multiple EC labels) are significantly more likely to be multi-domain proteins (Table 2). To quantify this association, a $\chi^{2}$ test was performed, yielding a highly significant association between multi-domain organization and multilabel enzymatic functions ($\chi^{2}=783.3$, $p<10^{-167}$). This result suggests that modular domain organization is associated with enzyme multifunctionality, indicating a bag-of-domains representation may enhance the modeling of the enzyme functions.

**Table 2:** Correlation between domain count and EC label count across enzymes in the training set.

	#domain = 1	#domain = 2	**#domain** > **2**
#label = 1	48 456	57 516	109 465
% per domain	95.7	93.3	95.1
#label = 2	2046	3916	4621
% per domain	4.0	6.4	4.0
#label>2	151	229	960
% per domain	0.3	0.4	0.8

### 3.2 Performance evaluation via cross-validation

The five-fold CV results are summarized in Table 1, available as supplementary data at *Bioinformatics* online, comparing our methods against the enhanced baseline, EnzHier (Duan *et al.* 2025). To ensure a fair comparison, we utilized the same 80% training and 20% test partitions previously established in the CLEAN and EnzHier studies. Since the original CLEAN study (Yu *et al.* 2023) reported CV performance comparable to EnzHier on the same training and test partitions, here we use EnzHier as the representative sequence-level baseline. Specifically, while EnzHier achieved a macro F1-score of 0.919, our MIL-BC model, which utilizes only the bag of DCT domain embeddings, reached a substantially higher F1-score of 0.962. This suggests that explicitly modeling the modular, multi-domain nature of enzymes is a more effective strategy than using a single, global sequence representation. Furthermore, the MIL-BC+ model, which augments the bag-of-domains embedding with a global protein embedding, achieved even higher accuracy (F1-score of 0.972), underscoring the robustness of our MIL framework.

### 3.3 Performance evaluation on independent datasets

To assess the generalization capability of our models, we performed a comprehensive benchmark on four independent test sets against several state-of-the-art methods (Table 3). DeepEC, CLEAN, and EnzHier are sequence-based methods, while CLEAN-Contact (Yang *et al.* 2024) is an extension of CLEAN that incorporates protein structural information generated by AlphaFold2 (Jumper *et al.* 2021). However, incorporating this structural component increases the total processing time, because AlphaFold2-based structure prediction and downstream structural processing (i.e. contact map) are computationally expensive. As a result, CLEAN-Contact is much slower than sequence-based methods.

**Table 3:** Evaluation of EC label prediction on four independent test sets.^a^

	**New-392**			**Price-149**			**Uniprot25-all**			**Uniprot25-multi**		
Method	Precision	Recall	F1	Precision	Recall	F1	Precision	Recall	F1	Precision	Recall	F1
DeepEC^b^	0.427	0.326	0.335	0.526	0.303	0.351	–	–	–	–	–	–
CLEAN	0.596	0.479	0.497	0.584	0.467	0.495	0.736	0.674	0.684	0.565	0.500	0.517
EnzHier	0.657	0.467	0.491	0.584	0.467	0.496	0.707	0.626	0.643	0.475	0.452	0.456
ESM2-BC	0.539	0.509	0.504	0.569	0.434	0.468	0.752	0.707	0.712	0.631	0.572	0.589
CLEAN-Contact	**0.663**	0.557	0.583	0.609	0.461	0.478	0.738	0.682	0.690	0.541	0.494	0.506
MIL-BC	0.552	0.519	0.519	0.570	0.507	0.509	0.728	0.701	0.696	0.669	0.602	0.611
MIL-BC+	0.629	**0.587**	**0.554**	**0.577**	**0.526**	**0.530**	**0.767**	**0.761**	**0.747**	**0.743**	**0.681**	**0.689**
MIL-CL+	0.634	0.523	0.541	0.557	0.487	0.486	0.729	0.695	0.698	0.536	0.506	0.511

a The best performances are highlighted in bold. Note that DeepEC, CLEAN, EnzHier, ESM2-BC, MIL-BC, MIL-BC+, and MIL-CL+ are sequence-based models, whereas CLEAN-Contact is a structure-based model.

b Results for DeepEC were taken directly from the published study (Kim *et al.* 2023), as the released code is no longer compatible with the latest Hugging Face framework; consequently, results on the two Uniprot25 test sets are unavailable.

Our MIL-based methods demonstrate superior performance, consistently matching or outperforming the global sequence embedding-based methods. On the New-392 and Price-149 datasets, the MIL-BC+ model achieved F1-scores of 0.554 and 0.530, substantially higher than those from the baseline models (e.g. EnzHier achieves the F1-scores of 0.491 and 0.496, respectively). Notably, MIL-BC+ outperformed MIL-BC across nearly all metrics. This indicates that the local domain and global protein features are complementary to each other, and explicitly modeling both of them significantly enhances the prediction. Surprisingly, the MIL-CL+ model, which aims to refine the latent representation of the MIL-BC+ model using CL, does not show further improvement (We further investigated CL with sigmoid inference in Table 5, available as supplementary data at *Bioinformatics* online). Although the precision score of MIL-CL+ increases to 0.634 on New-392 dataset, F1 score drops to 0.541 on the New-392 dataset. This trend was mirrored on the large-scale Uniprot25-all set, where MIL-BC+ (0.747 F1) again outperformed CLEAN (0.684 F1) and EnzHier (0.643 F1). Notably, the baseline ESM2-BC model achieved performance comparable to CLEAN and EnzHier, but fell short of the MIL models, indicating that the performance gains are primarily attributable to the MIL approach, rather than the transition from ESM-1b to ESM-2 global embeddings.

The most significant finding came from the Uniprot25-multi dataset, which specifically tests the difficult task of predicting functions for multi-label enzymes. On this set, sequence-level methods like CLEAN struggled significantly, achieving an F1-score of 0.517. In contrast, both the MIL-BC and MIL-BC+ models showed a substantial advantage, with MIL-BC+ achieving an F1-score of 0.689, a 33.3% improvement over CLEAN. This result validates our hypothesis that a bag-of-domains approach is critical for enhancing the prediction of EC numbers of multidomain proteins. However, MIL-CL+ underperforms relative to MIL-BC and MIL-BC+, suggesting that contrastive refinement does not consistently yield more discriminative representations for enzymes with complex or multiple functions. Increasing the number of training epochs for the CL component produced similar results, indicating that the observed performance gap is not attributable to insufficient optimization.

We further evaluated these models on two leakage-free test sets: Test-70 and Test-50, constructed to eliminate homologous overlap with the training data. As shown in Table 4, our proposed model MIL-BC+, achieves the best performance across all evaluation metrics on both leakage-free test sets. These results suggest that MIL-BC+ captures functionally relevant signals beyond simple sequence-level similarity, demonstrating improved generalization under stringent homology constraints.

**Table 4:** Evaluation of EC label prediction on leakage-free test sets.^a^

	Test-70			Test-50		
Method	Precision	Recall	F1	Precision	Recall	F1
CLEAN	0.619	0.537	0.552	0.564	0.476	0.491
EnzHier	0.623	0.495	0.516	0.568	0.425	0.446
ESM2-BC	0.623	0.549	0.564	0.541	0.465	0.481
CLEAN-Contact	0.656	0.575	0.588	0.598	0.511	0.526
MIL-BC	0.603	0.555	0.553	0.571	0.490	0.499
MIL-BC+	**0.668**	**0.617**	**0.611**	**0.637**	**0.551**	**0.555**
MIL-CL+	0.613	0.568	0.570	0.550	0.511	0.511

a Test-70 and Test-50 were constructed by enforcing sequence identity thresholds of 70% and 50%, respectively, against the training set. The best performances are highlighted in bold.

Table 2, available as supplementary data at *Bioinformatics* online, reports the F1 scores of the proposed MIL-based models in comparison with CLEAN and EnzHier for EC number prediction across different EC hierarchy levels. Across nearly all EC levels and all four evaluation datasets, MIL-BC+ achieves the best or near-best performance relative to the baseline models. Notably, MIL-CL+ demonstrates superior performance on the Price-149 dataset (L1-L3), indicating that CL can further enhance domain-level representations in certain data regimes.

### 3.4 Model interpretation using domain-level shapley additive explanation scores

One considerable advantage of the bag-of-domains MIL framework, compared with “black box” sequence-level models, is its inherent interpretability for the association between domains and their functions. To understand the model’s prediction, we compute a domain-level SHAP (SHapley Additive exPlanations; Lundberg and Lee 2017) value for each domain instance by aggregating the SHAP values across all dimensions of that specific domain’s embedding vector, which quantifies the marginal contribution of that entire domain to the final output. A large positive SHAP value for a given EC class indicates that the presence of that domain pushed the model’s prediction towards that specific function. In a biological context, this allows us to quantify the importance of the domain(s) most likely containing the active site or other critical functional determinants.

Figure 3 shows three examples from test sets. The protein with the UniProt ID P75442 (Fig. 3A and B) is correctly predicted to carry the functions of NaMN adenylyltransferase (EC number 2.7.7.18) and Ap4A hydrolase (EC number 3.6.1.41) by our MIL model. The UniProt record of this protein shows it has two domains (1–182, 191–344) responsible for the two functions, respectively. Our DCTdomain predicted two domains for this protein (shown in different colors in Fig. 3A), with boundaries (1–173, 174–344) closely matching those in the UniProt annotation. Based on the MIL-BC’s prediction, the first domain exhibits a high SHAP value (76.1) for the prediction of the first function (EC number 2.7.7.18), whereas the second domain exhibits a higher SHAP value (9.16 versus 0.71) for the prediction of the second function (EC number 3.6.1.41), indicating each of the two domains contributes to a distinct enzymatic function (Fig. 3B). In the second example Q8EGF8, the protein has two domains, but with one function (EC 2.7.7.65) presumably carried out by the second domain of this protein (shown in gray in Fig. 3C, with the putative active site and binding sites highlighted in ball-and-sticks in the 3D visualization). MIL-BC predicted a single function for this protein, with domain 2 contributing most strongly to this prediction according to the domain-level SHAP values (Fig. 3D). The results are consistent with UniProt annotation of this protein. In the final example Q40372 (Fig. 3E and 3F), although DCTdomain segments the protein into smaller subdomains (probably due to the missing contacts between the subdomains missed by the ESM-2 model), MIL-BC correctly predicted the function (EC 1.11.1.7) and the SHAP values suggested that domain 2 (containing residues 28–136, including the putative active site H69, proton acceptor highlighted in ball-and-sticks in Fig. 3E) contributed the most to the prediction. Together, these examples imply the MIL model can offer domain-level mechanistic explanations for the EC number prediction.

**Figure 3:**
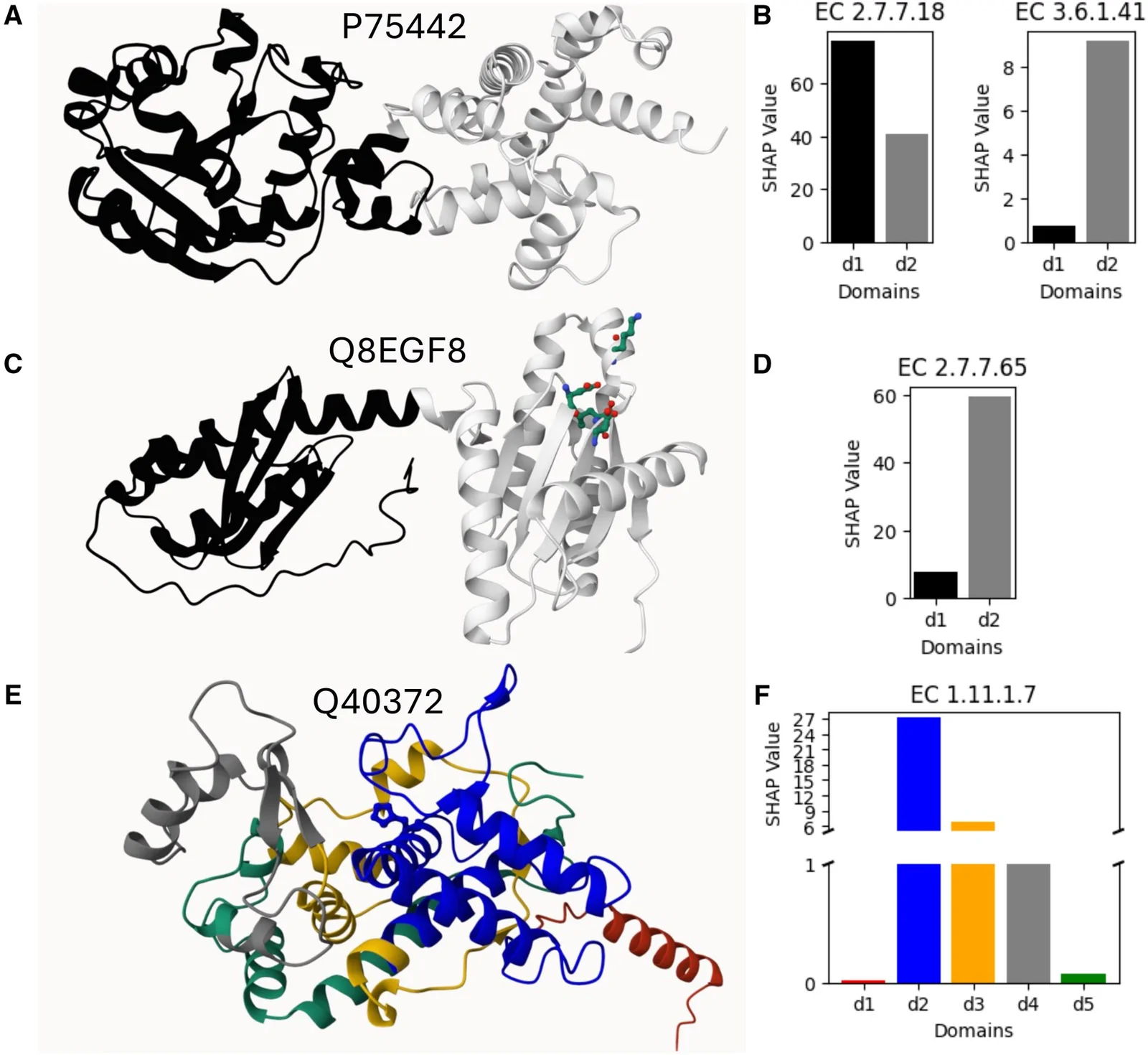
Examples of MIL-BC predictions and domain-level contributions to the predictions based on SHAP values. (A), (C), and (E) are the cartoon visualization of predicted 3D structures [by AlphaFold (Jumper *et al.* 2021)] of these proteins. Their domains, predicted by DCTdomain based on ESM-2 contact maps (not AlphaFold structures) are shown in different colors. Putative active sites and binding sites are highlighted in ball-and-sticks in (C) and (E). Molecular visualizations of the protein structures are prepared using PyMol (DeLano 2002). The barplots (B), (D) and (F) show the differential contributions (the magnitudes of the SHAP values) of the different domains to the predicted functions. In the barplots, the bars are shown in colors that match the corresponding domains in A, C, and E.

### 3.5 Ablation analysis

To isolate the contributions of individual architectural components, we conducted a detailed ablation analysis, with results summarized in Table 3, available as supplementary data at *Bioinformatics* online. We first evaluated the effect of incorporating Layer Normalization (LayerNorm) within both the instance-level and bag-level embedding networks. Across all evaluated datasets, the inclusion of LayerNorm led to consistent performance improvements, indicating enhanced training stability and representation quality. For example, on the Price-149 test set, the F1-score of the MIL-BC model increased from 0.493 to 0.513 when LayerNorm was applied.

We further examined the impact of label refinement, which enforces the assignment of at least one valid EC label by adjusting the BCE decision threshold. This refinement strategy improved predictive performance on the majority of test sets, as shown in Table 4, available as supplementary data at *Bioinformatics* online, suggesting that it effectively mitigates under-prediction in multilabel settings. Collectively, these ablation results highlight the importance of both layer normalization and label refinement in achieving robust and accurate EC prediction. We also investigated two inference methods on the same model checkpoint of MIL-CL+ in Table 5, available as supplementary data at *Bioinformatics* online, comparing sigmoid (used by MIL-BC+) with max-separation (used by CLEAN). It implies CL improved the representation when using sigmoid inference on New-392, Price-149, and Uniprot25-multi.

## 4 Discussion

Our results demonstrate that a Multiple Instance Learning framework based on a “bag-of-domains” representation is an effective strategy for enzymatic function prediction. While state-of-the-art methods like CLEAN have shown the power of CL on single, holistic sequence-level embeddings, our model’s performance suggests that this representation is insufficient. By treating an enzyme as a collection of functional instances rather than a single entity, our MIL-based models achieved superior performance, especially on the challenging multidomain multifunctional enzymes (e.g. in the Uniprot25-multi dataset).

The success of this framework is likely not limited to EC number prediction. We propose that this “bag-of-domains” approach serves as a general-purpose blueprint for predicting any protein function that is modular in nature. Many biological functions, such as fine-grained molecular activities (GO terms), protein–protein interactions, and viral taxonomic signatures, are mediated by localized functional domains rather than monolithic whole-sequence features. Because our multiple instance learning architecture decouples domain-level feature extraction from whole-protein aggregation, our MIL framework can be readily adapted to these problems, offering a more interpretable alternative to holistic sequence-based methods while remaining computationally tractable.

A key component of our current method is the *ab initio* partitioning of domains directly from the input sequence using the DCTdomain algorithm. While this is a flexible and powerful approach, it is still a predictive step that can introduce errors. The accuracy of our instances (the domain embeddings) is contingent on the accuracy of the contact map prediction and the RecCut segmentation. One direction for future improvement is to leverage the high-fidelity 3D structures (e.g. predicted by AlphaFold). By applying domain partitioning algorithms directly to these 3D structures, we could derive more accurate domain definitions. We hypothesize that using these “ground-truth” structural domains as instances for the MIL model would further enhance predictive accuracy.

## Supplementary Material

btag804_Supplementary_Data

## References

[btag804-B1] Ahmed N , Natarajan T, Rao KR. Discrete cosine transform. IEEE Trans Comput 1974;C-23:90–3. 10.1109/T-C.1974.223784

[btag804-B2] Bartlett GJ , Porter CT, Borkakoti N et al Analysis of catalytic residues in enzyme active sites. J Mol Biol 2002;324:105–21. 10.1016/s0022-2836(02)01036-7 12421562

[btag804-B3] Carbonneau MA , Cheplygina V, Granger E et al Multiple instance learning: a survey of problem characteristics and applications. Pattern Recognit 2018;77:329–53. 10.1016/j.patcog.2017.10.009

[btag804-B4] Claudel-Renard C , Chevalet C, Faraut T et al Enzyme-specific profiles for genome annotation: PRIAM. Nucleic Acids Res 2003;31:6633–9. 10.1093/nar/gkg847 PMC275543 14602924

[btag804-B5] Copeland RA. Enzymes: A Practical Introduction to Structure, Mechanism, and Data Analysis. John Wiley & Sons, 2023.

[btag804-B6] Davoudi S , Henry CS, Miller CS et al Ec-Bench: a benchmark for enzyme commission number prediction. Bioinform Adv 2026;6:vbag004. https://doi.og/10.1093/bioadv/vbag004 PMC12889163 41675694

[btag804-B7] DeLano WL. PyMol: an open-source molecular graphics tool. CCP4 Newsl Protein Crystallogr 2002;40:82–92.

[btag804-B8] Duan H , Li Z, Wu Y et al Enzhier: accurate enzyme function prediction through multi-scale feature integration and hierarchical contrastive learning. In: *International Symposium on Bioinformatics Research and Applications*. University of Helsinki, Finland: Springer, 2025, pp. 217–27.

[btag804-B9] Fruton JS. Proteins, Enzymes, Genes: The Interplay of Chemistry and Biology. New Haven, CT: Yale University Press, 1999.

[btag804-B10] Hirota K , Salim F, Yamada T. DeepES: deep learning-based enzyme screening to identify orphan enzyme genes. Bioinformatics 2025;41:btaf053. 10.1093/bioinformatics/btaf053 PMC11881691 39909853

[btag804-B11] Ilse M , Tomczak J, Welling M. Attention-based deep multiple instance learning. In: *International Conference on Machine Learning*. Stockholm, Sweden: PMLR, 2018, pp.2127–36.

[btag804-B12] Iovino BG , Tang H, Ye Y. Protein domain embeddings for fast and accurate similarity search. Genome Res 2024a;34:1434–44. 10.1101/gr.279127.124 PMC11529836 39237301

[btag804-B13] Iovino BG , Tang H, Ye Y. Protein domain embeddings for fast and accurate similarity search. In: International Conference on Research in Computational Molecular Biology (RECOMB’24). Cambridge, MA, USA: Springer, 2024b, pp. 421–4. 10.1101/gr.279127.124 PMC11529836 39237301

[btag804-B14] Jumper J , Evans R, Pritzel A et al Highly accurate protein structure prediction with AlphaFold. Nature 2021;596:583–9. 10.1038/s41586-021-03819-2 PMC8371605 34265844

[btag804-B15] Kanehisa M. Enzyme annotation and metabolic reconstruction using KEGG. In: Protein Function Prediction: Methods and Protocols. New York, NY: Springer, 2017, pp. 135–45. 10.1007/978-1-4939-7015-5_11 28451977

[btag804-B16] Kanehisa M , Goto S. KEGG: Kyoto Encyclopedia of Genes and Genomes. Nucleic Acids Res 2000;28:27–30. 10.1093/nar/28.1.27 PMC102409 10592173

[btag804-B17] Khosla C , Harbury PB. Modular enzymes. Nature 2001;409:247–52. 10.1038/35051723 11196653

[btag804-B18] Kim GB , Kim JY, Lee JA et al Functional annotation of enzyme-encoding genes using deep learning with transformer layers. Nat Commun 2023;14:7370. 10.1038/s41467-023-43216-z PMC10645960 37963869

[btag804-B19] Kim HR , Ji H, Kim GB et al Enzyme functional classification using artificial intelligence. Trends Biotechnol 2025;43:2214–31. 10.1016/j.tibtech.2025.03.003 40155269

[btag804-B20] Kumar N , Skolnick J. EFICAz2. 5: application of a high-precision enzyme function predictor to 396 proteomes. Bioinformatics 2012;28:2687–8. 10.1093/bioinformatics/bts510 PMC3467752 22923291

[btag804-B21] Li Y , Wang S, Umarov R et al DEEPre: sequence-based enzyme EC number prediction by deep learning. Bioinformatics 2018;34:760–9. 10.1093/bioinformatics/btx680 PMC6030869 29069344

[btag804-B22] Lin Z , Akin H, Rao R et al Evolutionary-scale prediction of atomic-level protein structure with a language model. Science 2023;379:1123–30. 10.1126/science.ade2574 36927031

[btag804-B23] Lundberg SM , Lee SI. A unified approach to interpreting model predictions. In: Advances in neural information processing systems, 2017, pp. 1–3. https://dl.acm.org/doi/10.5555/3295222.3295230

[btag804-B24] McDonald AG , Tipton KF. Fifty-five years of enzyme classification: advances and difficulties. FEBS J 2014;281:583–92. 10.1111/febs.12530 24103004

[btag804-B25] McDonald AG , Tipton KF. Enzyme nomenclature and classification: the state of the art. FEBS J 2023;290:2214–31. 10.1111/febs.16274 34773359

[btag804-B26] Morgat A , Lombardot T, Coudert E et al; UniProt Consortium. Enzyme annotation in UniProtKB using Rhea. Bioinformatics 2020;36:1896–901. 10.1093/bioinformatics/btz817 PMC7162351 31688925

[btag804-B27] Omelchenko MV , Galperin MY, Wolf YI et al Non-homologous isofunctional enzymes: a systematic analysis of alternative solutions in enzyme evolution. Biol Direct 2010;5:31. 10.1186/1745-6150-5-31 PMC2876114 20433725

[btag804-B28] Pearson WR. Protein function prediction: problems and pitfalls. Curr Protoc Bioinformatics 2015;51:4.12.1–8. 10.1002/0471250953.bi0412s51 26334923

[btag804-B29] Price MN , Wetmore KM, Waters RJ et al Mutant phenotypes for thousands of bacterial genes of unknown function. Nature 2018;557:503–9. 10.1038/s41586-018-0124-0 29769716

[btag804-B30] Rives A , Meier J, Sercu T et al Biological structure and function emerge from scaling unsupervised learning to 250 million protein sequences. Proc Natl Acad Sci USA 2021;118:e2016239118. 10.1073/pnas.2016239118 PMC8053943 33876751

[btag804-B31] Ryu JY , Kim HU, Lee SY. Deep learning enables high-quality and high-throughput prediction of enzyme commission numbers. Proc Natl Acad Sci USA 2019;116:13996–4001. 10.1073/pnas.1821905116 PMC6628820 31221760

[btag804-B32] Song Y , Yuan Q, Chen S et al Accurately predicting enzyme functions through geometric graph learning on ESMFold-predicted structures. Nat Commun 2024;15:8180. 10.1038/s41467-024-52533-w PMC11411130 39294165

[btag804-B33] Steinegger M , Söding J. MMseqs2 enables sensitive protein sequence searching for the analysis of massive data sets. Nat Biotechnol 2017;35:1026–8. 10.1038/nbt.3988 29035372

[btag804-B34] Tan JX , Lv H, Wang F et al A survey for predicting enzyme family classes using machine learning methods. Curr Drug Targets 2019;20:540–50. 10.2174/1389450119666181002143355 30277150

[btag804-B35] The UniProt Consortium, Uniprot: the universal protein knowledgebase in 2021. Nucleic Acids Res 2021;49:D480–9. 10.1093/nar/gkaa1100 PMC7778908 33237286

[btag804-B36] Vaswani A , Shazeer N, Parmar N et al Attention Is All You Need. In: *Advances in Neural Information Processing Systems*, Long Beach Convention Center, Long Beach, 2017. https://proceedings.neurips.cc/paper_files/paper/2017/hash/3f5ee243547dee91fbd053c1c4a845aa-Abstract.html

[btag804-B37] Yang Y , Jerger A, Feng S et al Improved enzyme functional annotation prediction using contrastive learning with structural inference. Commun Biol 2024;7:1690. 10.1038/s42003-024-07359-z PMC11666736 39715863

[btag804-B38] Yin Y , Mao X, Yang J et al dbCAN: a web resource for automated carbohydrate-active enzyme annotation. Nucleic Acids Res 2012;40:W445–51. 10.1093/nar/gks479 PMC3394287 22645317

[btag804-B39] Yu L , Tanwar DK, Penha EDS et al Grammar of protein domain architectures. Proc Natl Acad Sci USA 2019;116:3636–45. 10.1073/pnas.1814684116 PMC6397568 30733291

[btag804-B40] Yu T , Cui H, Li JC et al Enzyme function prediction using contrastive learning. Science 2023;379:1358–63. 10.1126/science.adf2465 36996195

