# Supplementary material for: Domain-aware enzyme classification via multiple instance learning
Source: Bioinformatics. 2026 Oct 30;42(11):btag804. doi: 10.1093/bioinformatics/btag804 (PMC13760209; doi:10.1093/bioinformatics/btag804)
Supplement: Supplementary file 1 [file btag804_supplementary_data.pdf]

Supplementary: Domain-Aware Enzyme Classification via Multiple Instance Learning

Table S1: Evaluation of MIL models compared with EnzHier under 5-fold cross-validation (using the same splits).

Method	Precision	Recall	F1
EnzHier	0.943	0.914	0.919
MIL-BC	0.961	0.969	0.962
MIL-BC+	0.966	0.984	0.972

Table S2: Comparison of EC prediction performance (F1 scores) across hierarchical levels L1–L4 (L1: X.-.-.-, L2: X.X.-.-, L3: X.X.X.-, L4: X.X.X.X), with the best scores highlighted in bold.

	New-392				Price-149				Uniprot25-all				Uniprot25-multi			
Method	L1	L2	L3	L4	L1	L2	L3	L4	L1	L2	L3	L4	L1	L2	L3	L4
CLEAN	0.944	0.907	**0.874**	0.497	0.940	0.916	0.808	0.495	0.933	0.895	0.876	0.684	0.946	0.871	0.851	0.517
EnzHier	0.912	0.883	0.801	0.491	0.888	0.880	0.795	0.496	0.880	0.833	0.820	0.643	0.875	0.793	0.782	0.456
ESM2-BC	0.863	0.836	0.813	0.504	0.820	0.804	0.712	0.468	0.894	0.883	0.865	0.712	0.879	0.836	0.800	0.589
MIL-BC	0.926	0.905	0.821	0.519	0.903	0.861	0.796	0.509	0.907	0.880	0.867	0.696	0.899	0.885	0.869	0.611
MIL-BC+	0.969	0.924	**0.861**	**0.554**	0.933	0.891	0.806	**0.530**	0.936	**0.903**	**0.888**	**0.747**	**0.948**	**0.918**	**0.893**	**0.689**
MIL-CL+	**0.970**	**0.938**	0.859	0.541	**0.954**	**0.927**	**0.832**	0.486	**0.938**	0.899	0.885	0.698	0.941	0.885	0.851	0.511

Table S3: Ablation study on input features and model components.

				New-392			Price-149		
	Input Features	LayerNorm	Label Refinement	Precision	Recall	F1	Precision	Recall	F1
MIL-BC	domain only	no	no	0.529	0.517	0.503	0.555	0.487	0.493
MIL-BC	domain only	yes	no	0.553	0.501	0.506	0.575	0.507	0.513
MIL-BC	domain only	yes	yes	0.552	0.519	0.519	0.570	0.507	0.509
MIL-BC+	domain + global	yes	no	**0.631**	**0.589**	**0.556**	0.565	0.513	0.518
MIL-BC+	domain + global	yes	yes	0.629	0.587	0.554	**0.577**	**0.526**	**0.530**

Table S4: Ablation study on the effect of label refinement across all four independent test sets.

		New-392			Price-149			Uniprot25-all			Uniprot25-multi		
Method	Label Refinement	Precision	Recall	F1	Precision	Recall	F1	Precision	Recall	F1	Precision	Recall	F1
MIL-BC	no	0.553	0.501	0.506	0.575	0.507	0.513	0.730	0.685	0.689	0.654	0.590	0.599
MIL-BC+	no	**0.631**	**0.589**	**0.556**	0.565	0.513	0.518	**0.768**	0.743	0.738	0.725	0.651	0.660
MIL-BC	yes	0.552	0.519	0.519	0.570	0.507	0.509	0.728	0.701	0.696	0.669	0.602	0.611
MIL-BC+	yes	0.629	0.587	0.554	**0.577**	**0.526**	**0.530**	0.767	**0.761**	**0.747**	**0.743**	**0.681**	**0.689**

Table S5: Ablation study on the inference methods across all four independent test sets. MIL-CL+ uses max-separation inference by default.

		New-392			Price-149			Uniprot25-all			Uniprot25-multi		
Method	Inference	Precision	Recall	F1	Precision	Recall	F1	Precision	Recall	F1	Precision	Recall	F1
MIL-BC+	sigmoid	0.629	0.587	0.554	0.577	0.526	0.530	**0.767**	0.761	**0.747**	0.743	0.681	0.689
MIL-CL+	sigmoid	0.624	**0.670**	**0.596**	**0.638**	**0.612**	**0.583**	0.693	**0.813**	0.722	**0.752**	**0.765**	**0.748**
MIL-CL+	maxsep	**0.634**	0.523	0.541	0.557	0.487	0.486	0.729	0.695	0.698	0.536	0.506	0.511
